# Chitosan immunomodulatory properties: perspectives on the impact of structural properties and dosage

**DOI:** 10.4155/fsoa-2017-0064

**Published:** 2017-09-14

**Authors:** David Fong, Caroline D Hoemann

**Affiliations:** 1 Institute of Biomedical Engineering, École Polytechnique, Montreal, QC, H3T 1J4, Canada; 2 Department of Chemical Engineering, École Polytechnique, Montreal, QC, H3T 1J4, Canada; 3 Bioengineering Department, George Mason University, Institute for Advanced Biomedical Research, Manassas, VA 20110, USA

**Keywords:** biomaterials, chitin, chitosan, inflammation, interferon, interleukin-1 beta, interleukin-1 receptor antagonist, macrophage

Due to its abundance, biodegradability and cytocompatibility, the polysaccharide chitosan has been investigated for use in a wide range of biomedical applications, including tissue engineering scaffolds [1,2], vectors for drug and gene delivery [3] and vaccine adjuvants [4]. Interestingly, chitosan exhibits considerable immunostimulatory activity, by inducing innate immune cells to release a wide range of pro- and anti-inflammatory cytokines, chemokines, growth factors and bioactive lipids [5]. Release of these mediators can have a significant impact on the performance of the different biomedical devices where chitosan is used. Despite considerable advances in our understanding of chitosan-innate immune responses *in vitro*, challenges persist in predicting whether a chitosan-based medical device will elicit pro- or anti-inflammatory responses *in vivo*. Addressing this knowledge gap is critical for a better and safer translation use of chitosan in biomedical devices. This paper will discuss important and recent advances in chitosan biology, and perspectives on the next unanswered questions.

Because chitosan is derived by chemical modification of a naturally sourced polymer, chitin, it is important to note that chitosan does not represent a single polysaccharide. Chitosan encompasses a family of linear polymers containing β-O-(1–4)-linked glucosamine (GlcN) and variable levels of N-acetyl glucosamine (GlcNAc). Deacetylation of chitin using enzymatic or chemical hydrolysis produces chitosan chains with anywhere from 40 to 100% GlcN content, also known as the degree of deacetylation (DDA) [3]. Following chemical deacetylation, GlcNAc residues are distributed in clusters (block acetylation); however, chitosans with homogenously arranged GlcNAc residues (random acetylation) can also be obtained by preparing a fully deacetylated chitosan that is then reacetylated to a particular DDA level [6]. In addition to the DDA and acetylation pattern, molecular weight (MW) is another important structural property that strongly influences chitosan biological and physicochemical properties [3]. Parent chitosan chains typically range from 200,000 to 1,000,000 weight-average MW; however, smaller average MW, even very small oligomers, can be targeted with enzymes or different acids that can specifically hydrolyze the β-O-(1–4)-linkages without substantially changing the DDA level [3,7–9]. The important point here is that there are a variety of possible approaches to produce chitosan, using chitin or chitosan from many different sources and suppliers. Many researchers have investigated chitosans with distinct DDA, MW, polydispersity, purity, form and dose, all of which can impact biological-innate immune responses. This has ultimately led to some confusion and divergent opinion as to whether chitosan is a pro-inflammatory or anti-inflammatory biomaterial.

Although immune-modulating properties of chitosan have been studied for over 30 years [10], intracellular chitosan response pathways have only been recently begun to be elucidated. Currently, the best-described intracellular signaling pathways involve cGAS-STING, and NLRP3 [4,11,12]. These pathways were shown to be deployed following macrophage exposure to chitosans with 80 to 98% DDA and 3 to 400 kDa in MW. The cGAS-STING pathway triggers a type 1 IFN response and specific downstream expression of the chemokine CXCL10/IP-10 [4,12]. Additionally, type 1 IFN responses, which signal through STAT1/STAT2 activation, induce the release of a well-known and therapeutically relevant anti-inflammatory factor, IL-1ra [12]. By contrast, activation of NLRP3 leads directly to inflammasome activation and to the release of pro-inflammatory factors IL-1β and PGE_2_ [11,12]. These two types of cytokine responses have been reported in both primary and cell line-derived human and mouse macrophage models as well as *in vivo*, providing great confidence that these responses to chitosan are reproducible and conserved across different species.

**Figure 1.**
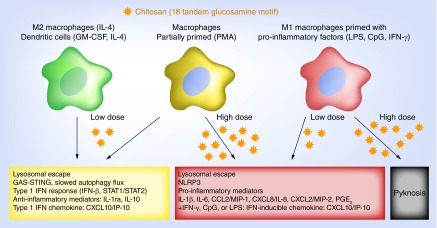
**Inflammatory responses to chitosan depend on prior immune cell activation state, chitosan dose, and the presence of a tandem glucosamine motif.** In macrophages primed with phorbol ester (PMA), low doses of chitosan induce type 1 IFN, leading to increased IL-1ra and CXCL10/IP-10 release and low levels of IL-1β and PGE_2_. High doses of chitosan activate the inflammasome, which leads to increased release of IL-1β and PGE_2_, and suppressed release of IL-1ra and CXCL10/IP-10. When macrophages are polarized towards an M1 state, chitosan enhances the release of pro-inflammatory cytokines. In macrophages polarized towards an M2 state, chitosan enhances the release of anti-inflammatory cytokines.

Most remarkably, these two signaling complexes were recently linked in the context of macrophage infection by intracellular pathogens such as *Mycobacterium tuberculosis*. In early intracellular pathogen infection, macrophages produce low levels of type 1 IFN, which in a paracrine manner induces the release of IL-1ra and IL-10 [13]. IL-10 suppresses the production of pro-inflammatory cytokines whereas IL-1ra antagonizes IL-1β autocrine signaling. This promotes host cell and thus pathogen survival by deceiving host immunity to downregulate pro-inflammatory signals that could result in cell death by pyknosis [13]. High multiplicity of infection can trigger strong inflammasome activation and high levels of PGE_2_. In this case, high levels of PGE_2_ suppress type 1 IFN, IL-1ra and IL-10 production [13]. Recently, we reported that a selected number of chitosans (3–10 kDa, 98% DDA; 10 and 190 kDa; 80% DDA, block-acetylated) in a particulate form mimic this model of macrophage infection by intracellular pathogens [12]. At low doses, chitosans containing the motif with a minimum 3000 Da consecutive GlcN residues (∼18 tandem GlcNs) induced a potent type 1 IFN response in macrophages without activating the inflammasome [12]. At higher doses, the same chitosans activated the inflammasome without eliciting a type 1 IFN response [12]. These interesting findings suggest that macrophages use the same sensing mechanisms to produce anti-inflammatory signals after chitosan particle or intracellular pathogen uptake. Lysosomal escape was found to be the novel and common mimetic event (Figure 1) [12]. While it has been reported that chitosan intrinsic properties play a significant role on the release of pro- or anti-inflammatory signals [14–16], the impact of dose was only recently recognized. This newly identified effect of dose highlights the potential to fine-tune chitosan use, not only in terms of structural properties, but also dose, to elicit or avoid specific immunomodulatory responses for a desired biomedical application.

When interpreting findings from the literature, there are many variables related to the experimental design that may drastically influence whether specific cytokine responses to chitosan stimulation are observed. These include properties of the chitosan preparation (i.e., DDA, MW, polydispersity), chitosan dosage, the cellular model used (e.g., primary or cell line, murine or human origin) and the presence of pro-inflammatory costimulation agents (e.g., LPS, CpG or IFN-γ) [4,5]. For instance, IL-1β production in macrophages following chitosan stimulation is routinely observed in macrophages primed with CpG or LPS [16–18], provided that the chitosan contains at least 3000 Da consecutive GlcN monomers, and moderate chitosan doses are used (∼50 μg.ml^-1^) [12]. When macrophages are treated with very high doses of chitosan, cell pyknosis is observed (Figure 1) [19]. These sources of variability emphasize why it is crucial to fully characterize the chitosan intended for use in biological studies. Far too often, the chitosan used have either been partially characterized for DDA or MW (i.e., DDA determined to be above or below a certain value, or MW between certain range with no average value or polydispersity index), or characterized thoroughly for only one of the two properties, leaving the other properties unknown [5]. Endotoxin content, which is rarely reported in the chitosan preparation, can also confound data interpretation.

The critical effect of chitosan dose *in vitro* on differential cytokine induction by macrophages (i.e., induction of a type 1 IFN response vs activation of the inflammasome) begs the question of what happens *in vivo* and more specifically how chitosan dose influences immune cell cytokine production *in vivo*. There are multiple attributes from the *in vivo* setting that the *in vitro* scenario cannot reproduce. For one, macrophage, neutrophil, mast cell and dendritic cell monoculture models do not consider the involvement of other immune cells. Yet, it is well known that a variety of innate immune cell types are elicited by chitosan, interact among each other and contribute in different ways to the host response to chitosan. Next, molecules in the extracellular environment and cell-to-cell interactions are largely simplified *in vitro*, and it is likely that this can influence immune cell response to chitosan. For instance, chitosan can interact with serum proteins, and this significantly affects how chitosan is recognized and presented to cells, which can potentially have an important impact on host response to chitosan [20]. Measuring the effects of chitosan dose and structure in an *in vivo* setting will be important to pursue and will provide valuable insights on host responses to chitosan.

Chitosan is the only natural cationic polysaccharide and has easily tunable properties that make it an attractive material for biomedical applications. Its immunomodulatory effects are rather wide, as chitosan can induce, based on structural properties and dosage, a plethora of cytokines of pro- or anti-inflammatory nature. Whether these pro- or anti-inflammatory responses are good or bad ultimately depends on the context. The precise difficulty in saying ‘chitosan is pro-inflammatory’ is that ‘inflammation’ covers a very broad range of cellular and molecular responses that in fact can be beneficial or harmful depending on the type and degree of inflammation. Inflammatory cell infiltration is necessary and beneficial for wound repair, but excessive cell infiltrate accompanied by classical immune cell activation can result in tissue damage. Inflammatory responses to chitosan must be evaluated with the perspective of how cellular, molecular and tissue responses are expected to influence the safety and performance of the biomedical device. Structure–function models are being established to predict both release of pro- or anti-inflammatory cytokines from immune cells *in vitro*. Developing relevant models *in vivo* will be critical to designing biomaterials and devices containing chitosan that capitalize or minimize specific inflammatory responses.
